# Fluorescence Microscopic Investigations of the Retarding Effect of Superplasticizers in Cementitious Systems of UHPC

**DOI:** 10.3390/ma13051057

**Published:** 2020-02-27

**Authors:** Johannes Arend, Alexander Wetzel, Bernhard Middendorf

**Affiliations:** Department of Structural Materials and Construction Chemistry, University of Kassel, Mönchebergstr. 7, 34125 Kassel, Germany; alexander.wetzel@uni-kassel.de (A.W.); middendorf@uni-kassel.de (B.M.)

**Keywords:** UHPC, superplasticizer, retardation, fluorescence microscopy

## Abstract

The adsorption of superplasticizer molecules to particle surfaces in cementitious systems is a very important aspect for the desired liquefaction of pastes and concretes. This way, the comb shaped polymers shield attractive forces between the particles and induce a well-dispersed, homogeneous suspension. These admixtures allow the usage of fine fillers even in combination with low amounts of mixing water, and thus, are the basis for modern high performance concretes. However, the adsorption does not cause beneficial effects only: The polymer covered particle surfaces, especially clinker, are hindered to interact with water, thus hydration is retarded. This is the reason for lower early strength and is very disadvantageous for certain applications. Today it is known that the molecular structure of the polymers, for instance the chain length and charge density, affects the retardation strongly. The complexity and diversity of cementitious systems is the main reason why research in this field is quite empiric and time as well as cost intensive. To investigate the adsorption of superplasticizers in various systems in-situ, a fluorescence microscopic approach was applied: By staining the polymers with fluorescent dye they become localizable and the adsorption quantifiable. This work shows the influence of molecular structure to adsorption characteristic of different polymers and the correlation to the retarding effect of superplasticizers, especially concerning the presence of silica fume, which is indispensable for ultra-high performance concrete (UHPC).

## 1. Introduction

The enhanced properties of ultra-high performance concrete (UHPC), such as compressive strength (>150 MPa), durability and chemical resistance, are strongly connected to modern organic admixtures—so-called superplasticizers (SPs) [1,2,3]. The only way to achieve the unique characteristics of UHPC is to generate a very dense cementitious matrix by combining a low water/binder ratio (w/b) of about 0.2 with the usage of micro to nano fine fillers like silica fume. Without chemical admixtures this combination causes strongly agglomerated and heterogenic mixtures, unusable for application. To overcome these challenging requirements - extremely high specific surface of solids cause high water demand but low water content for low porosity -superplasticizers are essential to obtain a workable consistency of fresh mortar or concrete. The invention of polycarboxylate ether (PCE) superplasticizers in 1981 by Hirata [4] promoted a huge step in the development of cementitious construction materials because these polymers showed superior potential to plastify mixtures with low water content and very fine mineral compounds. In comparison to former plasticizers such as lignin sulfonates or polycondensates [5], PCEs are comp-shaped polymers with a polycarboxylate backbone carrying polyether sidechains. This molecular structure causes two complementing effects: The carboxyl groups of the backbone are deprotonated in cement typical pH range (~12.6) and thus negatively charged [6]. In aqueous suspensions, mineral particles show surface charges depending on several experimental parameters such as pH-value and electrolyte composition [7]. Beside van der Waals forces, this phenomenon is the basis for an electrostatic interaction between the charged polycarboxylate backbone and the surface of the particles, resulting in an adsorption of the polymers [8]. This way, on the one hand, electrostatic attraction between the particles are shielded, on the other hand, the sidechains cause steric repulsion between polymer-covered particles. These two effects prohibit an agglomeration and allow a homogeneous dispersion of mineral fines within a system with low w/b ratio and a workable mixture is obtained. Nevertheless, the use of PCE superplasticizers does not only come along with beneficial aspects: The adsorption of the polymers to the mineral surfaces causes a passivation of reactive phases especially of the cement, because water is hindered to reach the clinker phases. Thus, dissolution and resulting from that cement hydration is inhibited. In addition, not adsorbed PCE is able to interfere the precipitation of calcium silicate hydrate phases (C–S–H) because calcium ions (Ca^2+^) tend to be complexed by carboxyl groups of the PCE [9]. Similar retarding phenomena are observed if heavy metals are present in the cement mixture, because Ca^2+^ is consumed by the precipitation of insoluble salts [10,11]. Depending on the application, this retardation is a big issue for example in terms of production speed and formwork usage. As well as the plasticizing effect, this retardation depends strongly to the adsorption and the chemical structure of the superplasticizers [12]. Because cementitious systems are quite diverse depending on the used materials and application [13], general assumptions of admixture effects are limited. Therefore, empiric studies are necessary to find the optimal mixture for certain applications. To reduce the effort of testing, current research is trying to establish prediction methods for the plasticizing and retarding effect of PCEs. Marchon et al. showed impressively how changes in the molecular structure of classic PCEs influence their performance in terms of plastification and retardation [14]. Furthermore, the theoretical prediction of an optimal superplasticizer structure (best plasticizing effect without enhanced retardation) by analyzing experimental data in special plots succeeded for a model cement clinker [15]. Another approach by Lange et al. established a correlation between the so-called HLB-value (hydrophilic–lipophilic balance) and the effects of PCEs to the rheological properties of cement and concrete pastes [16,17]. Beside these examples, a lot of research is in progress to optimize superplasticizers for various applications and (blended) cements, for example in terms of robustness and effectiveness [18,19,20,21,22,23]. Because of the high diversity of cementitious systems and potential structures of superplasticizers, general simulating approaches are not established yet and will probably be limited in the future as well. On the other hand, the experimental effort and material usage of empiric studies should be avoided as far as possible to ensure purposeful research. In this context, in-situ methods are helpful to investigate the interaction of superplasticizers with mineral particles and within cementitious mixtures with a time resolution of seconds or minutes. The fluorescence microscopy provides the ability to localize superplasticizer polymers in suspensions in real time. The correlation of microscopic data to results obtained with other methods allows new insights for superplasticizer science and was presented by Arend et al. [24]. In the present work, the in-situ-method was applied for the investigation of the retarding effect of two different PCEs in a cementitious model system. Due to the characteristics of a relatively high dosage of PCE and the presence of silica fume, which strongly affects the performance of superplasticizers in general [21], the findings of this experimental setup can be correlated with retardation effects in UHPC. The hydration of cement pastes with and without silica fume was investigated by heat flow calorimetry varying the type and dosage of two different PCE superplasticizers. Results gained by fluorescence microscopy as described in [24] were correlated to the calorimetric investigations of the hydration within the first hours. To find the answer to why the superplasticizers retard the investigated mixtures in a different way, the cementitious system was modified to allow the localization of the polymers by fluorescence microscopy which enables real time investigation of the dynamic hydration process of cement. The approach, modifications and results are presented and discussed in the following sections.

## 2. Materials and Methods

### 2.1. Solids

This work is focused on the hydration of an ordinary Portland cement (OPC), Type CEM I 52,5 HS/NA (Holcim, Hamburg, Germany) acc. DIN EN 197-1, which is primary used in UHPCs developed in the German priority program 1182 funded by the Deutsche Forschungsgemeinschaft (DFG) [25], especially for the so-called M3Q mixture. The phase composition determined by x-ray diffraction analysis (XRD) and Rietveld refinement is given in Table 1. As silica fume, *silicol P* (Sika, Baar ZG, Switzerland) was used.

The results of the x-ray fluorescence analysis (XFA) for the used cement and the silica fume are shown in Table 2.

### 2.2. Superplasticizers

**Figure 1:**
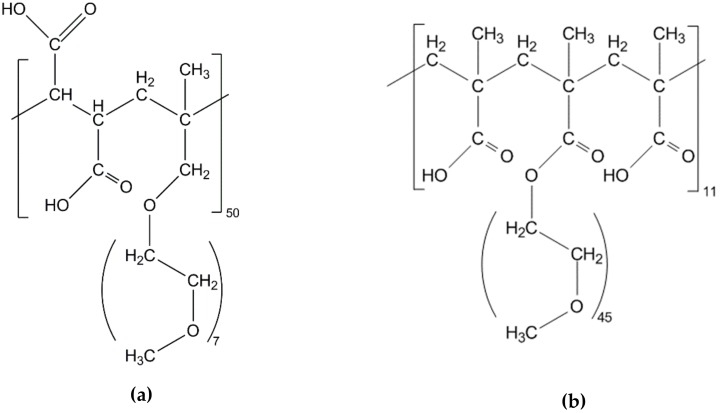
Chemical structure of (**a**) APEG and (**b**) MPEG.

The superplasticizers were synthesized and characterized by the research group of Prof. Plank at the Department of Chemistry at Technical University Munich, Germany. The first one is an allyl ether based comb polymer containing maleic anhydride monomers (APEG, Figure 1a). In the synthesis the allyl ether monomer does not homopolymerize due to mesomeric stabilization, thus the polymer shows a strictly alternating monomer structure of side chain and charge carrying groups. The side chains with just seven ether units are rather short compared to usual MPEG-type PCEs. The maleic anhydride monomer induces two carboxylic groups in the repeating unit right next to each other, causing a high charge density of the polymer [26]. The second PCE is a so-called MPEG-type one and consists of randomly polymerized meth acrylic acid and methoxy polyethylene glycol. In this work an agent with a monomer ratio of six meth acrylic acids to one polyethylene glycol is used, representing long side chains of 45 ether units (MPEG, Figure 1b) [27]. It is often specified as 45PC6 because of these ratios of chemical groups, but in this work just MPEG is used as label. The superplasticizers were obtained as solutions in water with a polymer content of 35 wt% in case of APEG and 40 wt% of MPEG. The superplasticizer dosage in UHPCs is in general quite high in comparison to other systems because of an overall very high specific surface of the fine materials and the presence of silica fume which is known to strongly interact with PCE polymers [21]. In this work two PCE dosages representing a spectrum used in UHPCs are investigated, which are given in percent by weight of binder (%bwob): 0.5 %bwob and 1.2 %bwob.

### 2.3. Calorimetry

The most common way to investigate the hydration of cement is to measure the heat flow that is produced by the physicochemical reaction. Therefore, samples with varying silica fume to cement ratio, superplasticizer-type and superplasticizer content were investigated. If superplasticizers are used, the w/b ratio was kept at 0.25 and the water content of the superplasticizer solution was considered in the total amount of mixing water. As reference, samples without superplasticizers were mixed. To enable a homogenous mixture, the w/b ratio was 0.4 in this case. The detailed sample composition is shown in Table 3. The isothermal calorimeter MC-CAL/100P (C3, Haar, Germany) was used for the heat flow measurements at the temperature of 20 °C.

### 2.4. Staining

For the staining process a chemical procedure, which requires an organic solvent (toluene) for the staining reaction as well as a two-step reaction for binding the rhodamine B-dye to the polymers, was used [24]. By using amino-fluorescein (Merck, Darmstadt, Germany) it is possible to bind the fluorescence dye fluorescein in aqueous conditions directly to the carboxylic groups of the polymers. Therefore, the carboxylic groups were activated with 1-ethyl-3-(3-dimethylaminopropyl)-carbodiimide hydrochloride (EDC; Merck, Darmstadt, Germany) enabling the reaction with amino groups to amide bonds [28]. Starting from the PCE-solution, the different polymer contents were considered and a total mass of 5.7 g APEG- and 5.05 g MPEG-solution were used to have 2 g of each polymer in a round bottom flask. The PCE-solutions were diluted with MES-buffer-0.1 M 4-morpholinoethanesulfonic acid (Merck, Darmstadt, Germany) and 0.5 M sodium chloride in water-to a mass of 48 g per reaction mixture. 5.2 mg amino-fluorescein (0.015 mmol) were dissolved in 1 mL of dimethyl sulfoxide (DMSO). After the dye dissolved completely, this mixture was added to the diluted polymer solution. To start the reaction, 28.8 mg EDC were dissolved in 1 mL MES-buffer and were added as well after complete solution of the solid. The reaction mixture was stirred for 96 h at room temperature, before the product was purified by dialysis. Therefore, the reaction mixture was filled in dialysis tubes with a MWCO of 3.5 kDa (Carl Roth, Karlsruhe, Germany) and stored in deionized water under stirring. This way, the large polymer molecules were hold inside the tubes while unbound dye, side products and buffer substances pass the permeable membrane. The washing water was replaced until no coloration could be observed. The purified solution of stained polymer was dried via rotary evaporator, obtaining a slightly yellow solid. When mass stability was reached after vacuum drying overnight, the PCE-solid could be used for the microscopic experiments. It is essential for the fluorescence microscopic approach to be sure that the dye is chemically bound to the polymers and the fluorescence signal is proportional to the local superplasticizer concentration. Therefore, fluorescence spectroscopy was applied to show a bathochromic shift of the excitation- and emission peak, which is characteristic for a dye molecule bound to a bigger structure [29]. 1 mg stained MPEG was dissolved in 0.01 M potassium hydroxide solution (1 mL) to be analyzed with the fluorescence spectrometer CLARIOstar (BMG Labtech, Ortenberg, Germany). For comparison, 1 mg solid untreated MPEG was dissolved in a potassium hydroxide solution (0.01 M) containing 0.1 mmol amino-fluorescein, representing the case of an unreacted polymer-dye-mixture. If such peak shifts can be detected, the chemical bonding between dye and superplasticizer-polymer is proved.

### 2.5. Fluorescence Microscopy

With the used fluorescence stereoscope M 205 FA (Leica Microsystems GmbH, Wetzlar, Germany) and the sample holder described in [24] diluted suspensions can be investigated. Besides the fluorescence mode, the stereoscope is equipped with normal light mode as well. To enhance the contrast of the images, only coarse particles of the used CEM I 52,5 were separated by washing with iso-propanol followed by decantation and drying. The mineral composition was checked via XRD and showed no deviation to the original cement. In order to obtain images of separated particles, the resulting w/b ratio for these experiments was 5. To compensate the low solid concentration, synthetic pore solution [30] was used as liquid phase to allow clinker hydration even in this diluted cementitious system. On the one hand, samples of 10 mg coarse cement particles were mixed with 50 µL pore solution with a PCE content of 1 mg/mL resulting in a superplasticizer to binder ratio of 0.5 %bwob. The second batch of samples consisted of 8.5 mg coarse cement and 1.5 mg of silica fume with the same PCE solutions. The suspensions were intensively mixed by shaking in an Eppendorf tube. 3 µL of these suspensions were placed between the microscopic slides of the sample holder. As fast as possible (approx. one minute after mixing), the investigation was started and during 6 h every 15 min a fluorescence image was taken. For the APEG samples, the illumination time was set to 1 s; for MPEG samples it was 3 s to compensate a lower overall fluorescence intensity.

## 3. Results and Discussion

### 3.1. Calorimetry

**Figure 2:**
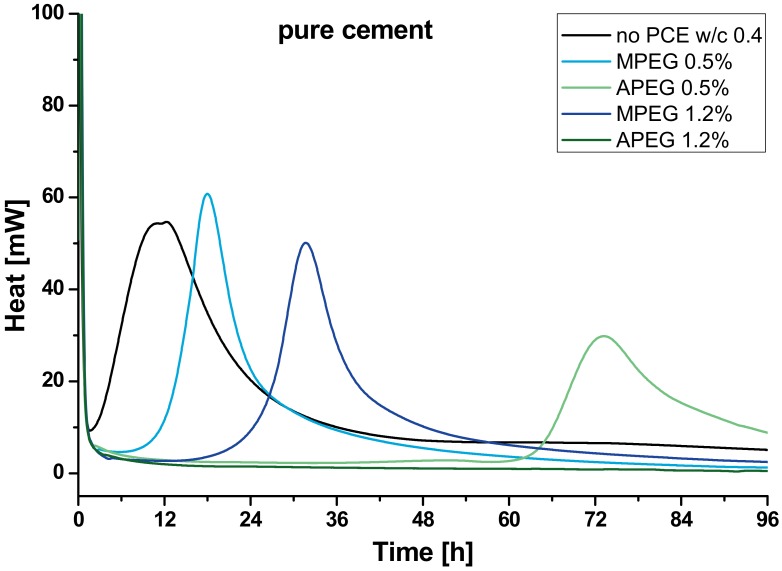
Heat flow of samples with pure cement (CEMI 52,5 HS/NA, black) with 0.5 %bwob MPEG (light blue), 0.5 %bwob APEG (light green), 1.2 %bwob MPEG (dark blue) and 1.2 %bwob APEG (dark green).

The heat flow during the first four days after mixing of the pure cement samples with APEG and MPEG in different dosages (Table 1) are shown in Figure 2.

The investigated cement shows two almost overlapping hydration peaks, while for samples with PCE only one is indicated. In general, the first peak of pure cement’s heat flow is caused by the precipitation of C–S–H; the second is due to the formation aluminate phases [14]. Depending on the dosage and used PCE the hydration of the samples is retarded or in case of 1.2 %bwob APEG completely inhibited. MPEG retards the reaction 6 h (0.5 %bwob) and ~24 h in case of 1.2 %bwob dosage. If 0.5 %bwob APEG is used, hydration is retarded for about 60 h.

The calorimetrical results point out a clear dependency of the hydration retardation to the used PCE. The higher the PCE is dosed, the more the hydration is retarded. For common dosages of superplasticizers (>0.5 %bwob) this finding stands in line for example with work by Zingg [31] and is comprehensible, concerning the working mechanism of the polymers, adsorbing to, and thus covering, the clinker surface. The extreme retardation of APEG culminating in a complete suppression of the hydration in combination with the used cement is quite surprising and not described in literature yet. Although such high dosages of APEG have not been investigated in terms of the rheological effect thus far, it is a good subject to study correlations to fluorescence microscopic results.

**Figure 3:**
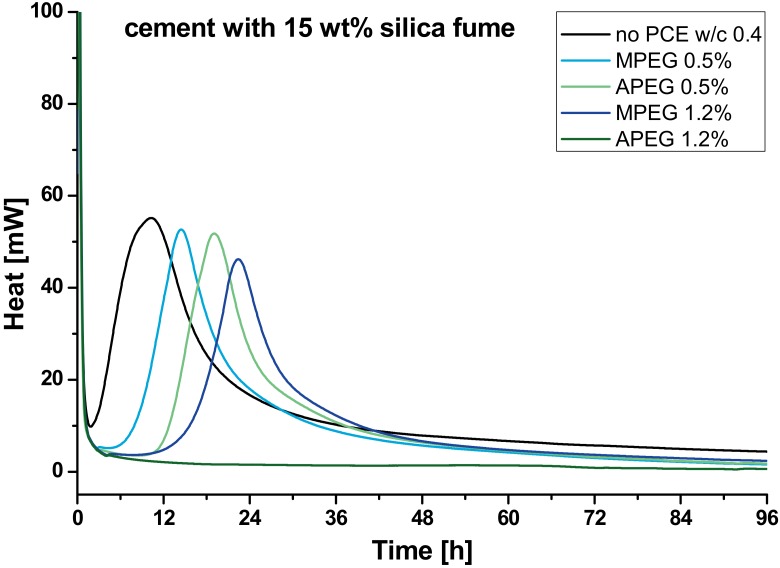
Heat flow of samples with cement and 15 wt% silica fume (black) with 0.5 %bwob MPEG (light blue), 0.5 %bwob APEG (light green), 1.2 %bwob MPEG (dark blue) and 1.2 %bwob APEG (dark green).

In Figure 3 the results of the calorimetry of the samples with silica fume (Table 3) are shown.

In the presence of silica fume, the two hydration peaks turn to a main peak with a little shoulder. Again, samples with PCE show only one peak. The presence of silica fume reduces the retardation remarkably in four of five cases: Without any polymer, the hydration peak of the sample reaches the maximum about two hours earlier than pure cement. For 1.2 %bwob MPEG the time reduction is quite significant, but especially for 0.5 %bwob APEG it is remarkably reduced by more than 50 h. In contrast, the hydration is still completely inhibited if the APEG dosage is 1.2 %bwob. With the addition of silica fume, the total specific surface of the system increases. This issue will be discussed in detail in Section 3.3.

### 3.2. Fluorescence Spectroscopy

**Figure 4:**
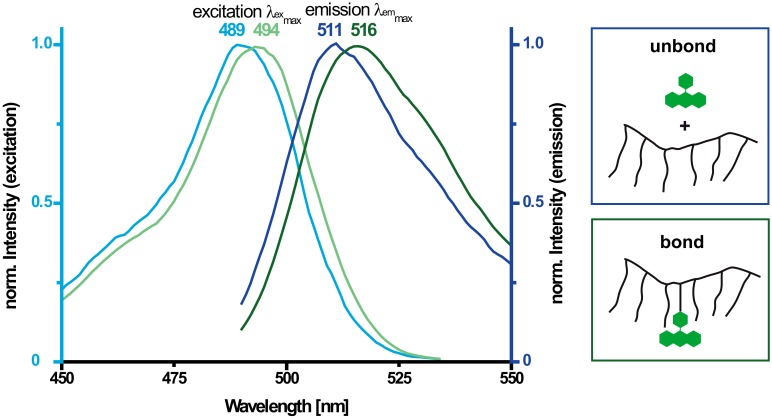
Spectra of excitation (light) and emission (dark) of stained MPEG (green) and unreacted PCE-dye-mixture (blue) in 0.01 M potassium hydroxide solution.

For better illustration of the peak shifts, the signal intensities of the excitation and the emission spectra in Figure 4 are standardized to 1. The maximum of the excitation peak for free amino-fluorescein mixed with SP-polymer is reached at 489 nm and the emission maximum at 511 nm. The spectra of the bound amino-fluorescein in the stained MPEG show bathochromic shifts of 5 nm, thus the maximal excitation is reached at 494 nm and the emission maximum at 516 nm, indicating a chemical bonding of dye and superplasticizer [29].

### 3.3. Fluorescence Microscopy

#### 3.3.1. Pure Cement

**Figure 5:**
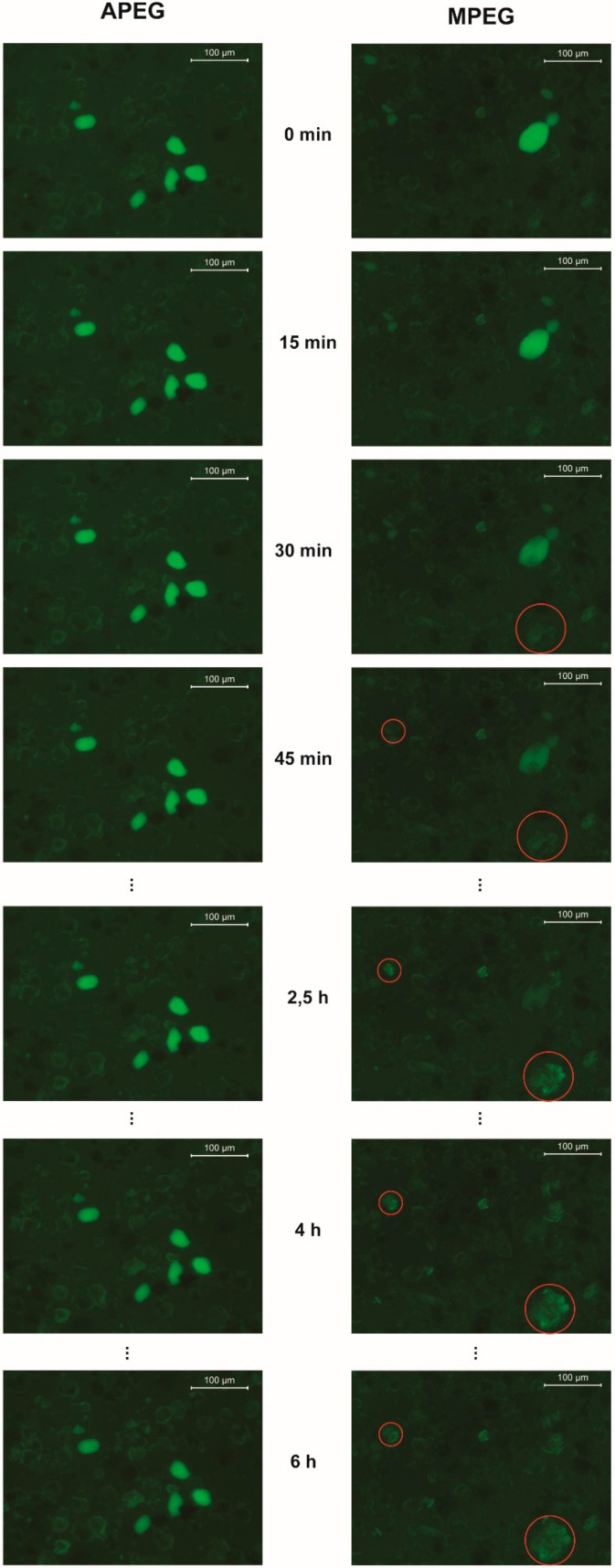
Fluorescence images of cement suspensions in synthetic pore solution with APEG (left) and MPEG (right) in a time period of 6 h. The red circles mark growing hydrate phases.

The microscopic images of the samples with stained APEG and MPEG show different kinds of mineral particles: Electron microscopic investigation of the coarse cement showed sharp, broken particles (clinker) and rounder ones, which are characterized by energy dispersive x-ray spectroscopy (EDX) to be calcium sulfate. Via XRD analysis, both gypsum and hemihydrate were detected, but within the fluorescence microscopic experiment, both could not be differentiated. Thus, these particles are called gypsum in the following, although it cannot be excluded that hemihydrate particles were investigated as well. The strong fluorescence signal of the round particles indicates a high amount of adsorbed PCE, while the adsorption on the clinker particles is much more heterogeneous. The time resolved image series (Figure 5) indicate a very different dissolving behavior of the gypsum particles depending on the used PCE. In the investigated time period of 6 h, no significant changes in the fluorescence images of the APEG sample can be noticed and the particle morphology did not change. On the other hand, the gypsum dissolves in presence of MPEG completely in about 3 h and hydration products appear over time (marked with red circles in Figure 5).

**Figure 6:**
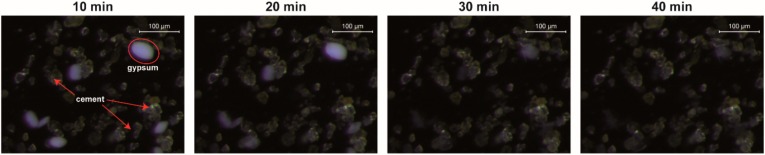
Light microscopic images of cement suspension in synthetic pore solution without PCEs. White gypsum particles dissolve in 30–40 min.

The gypsum particles are identifiable by color and shape in light microscopy as well, thus, measurements without PCE (just coarse cement in pore solution) were made to investigate the dissolving speed. For the used experimental parameter, the gypsum dissolves fast and the white particles disappear within 30–40 min (Figure 6).

Considering the proven bound of the dye to the PCEs, the most polymer adsorbs in case of pure cement to the gypsum particles shown by the bright signal in the fluorescence images for these experimental conditions. It is known that SP interact strongly with very early hydrate phases such as ettringite, which forms rapidly by the reaction of C_3_A with sulfate [14]. In the microscopic experiments, a very fast (within minutes) precipitation of such phases in noticeable amounts was not detected by changes in the fluorescence images. PCEs should have adsorbed to these phases and caused remarkable fluorescence signals, thus, C_3_A-surfaces seem to be passivated. At this point it is not clear, if this passivation is based on immediate precipitation of ettringite after mixing cement and PCE-solution, before the measurement could have been started, or by a PCE-coverage. On the one hand, this issue should not have major influence on the experiment because of the low C_3_A-content of the used cement, but on the other hand, Figure 5 indicates at least a time dependent connection between the dissolution of the gypsum particles and the formation of hydrates. An explanation could be that the concentration of sulfate for ettringite precipitation is not sufficient until the sulfate from the gypsum particles dissolves, but because synthetic pore solution with an already high sulfate content is used, this is not plausible and a passivation of the clinker phases is more obvious.

However, although often investigated in terms of early hydration, e.g., by Stroh [32], the formation of ettringite does not represent the hydration of the silicate phases and thus the retarded hardening of the cement, but nevertheless the fluorescence microscopic findings are quite remarkable. It is not clear if the observed formation of hydrate phases (MPEG, >30 min) is due to the presence of PCE or the experimental setup: owed to the low solid content in suspension, the lack of dissolved ions was tried to be compensated with synthetic pore solution. In any case, the fact that in case of APEG experiments no hydration products and dissolution of gypsum were observed is a clear evidence for the different retarding effects of the PCEs.

To take the chemical structure of APEG and MPEG into account, the main differences are the chain lengths and the charge density of the backbone as a result of the different polymerized monomers. In literature, the charge density of APEG is given as 3700 µeq/g at a pH value of 13 [26], while MPEG has 1600 µeq/g [9] at comparable conditions. A conclusion just from these values, that APEG adsorbs stronger to surfaces just because of the higher charge of the polymer resulting in a denser layer on the reactive phases and thus causing more retardation, may fall short. Although in general an increased charge leads to increased adsorption [33], the chemical conditions need to be considered. If dissolved calcium ions are present, the charge density of both polymers drops massively because the charge carrying carboxyl groups are widely complexed by the dications (Ca^2+^) [9]. In this case, the charge density of APEG is given as 300 µeq/g [26] and MPEG about 600 µeq/g [9], thus, the order is inverted. Nevertheless, the fluorescence microscopic experiments indicate the most adsorption at the gypsum particles, which is unexpected because gypsum is reported to interact with PCEs not appreciably [33]. However, if the dissolution of the gypsum is an indicator for the polymer barrier between soluble or reactive surfaces and water (no PCE < MPEG < APEG), it correlates with the retardation of the investigated systems. The effect of silica fume to accelerate the hydration in presence of the PCEs (3.1) supports this interpretation because the specific surface, which adsorbs polymer as well [34] and promotes nucleation of hydrate phases, is increased significantly, while the amount of PCE is constant. Thus, the amount of polymer per surface decreases, the polymer barrier becomes thinner and retards the hydration less. Only in case of the high dosage of APEG (1.2 %bwob) this effect is not observed. APEG seems to form a strong barrier with less polymer required than MPEG. In contrast to the dosage of 0.5 %bwob for both superplasticizers, there is enough polymer to cover even the silica fume surface additionally to the cement.

#### 3.3.2. Cement with Silica Fume

**Figure 7:**
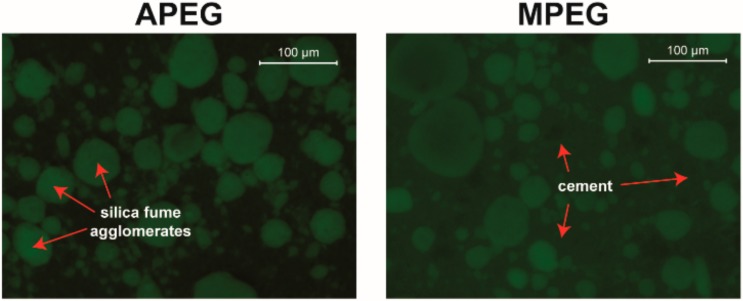
Fluorescence images of cement/silica fume suspensions in synthetic pore solution with APEG (left) and MPEG (right) 15 min after mixing.

Although the suspensions of cement with silica fume were mixed carefully prior to the measurements, the images are dominated by round agglomerates of silica fume with sizes up to 100 µm, and cement particles are hard to identify. Moreover, the images are somewhat diffused as a consequence of dispersed silica particles, which cannot be resolved by the stereoscope. Nevertheless, a very strong adsorption of both PCEs to the silica fume is indicated by the bright fluorescence of the agglomerates (Figure 7). In case of APEG, the higher contrast indicates even more adsorbed polymer than in case of MPEG, because a higher amount of dispersed and thus not clearly detectable silica particles resulting in a stronger background signal (as seen in Figure 7, MPEG) is not expected for the same sample preparation.

In contrast to the measurements of the cement suspensions without silica fume, no significant changes of the fluorescence images over time could be noticed.

The reason why no time dependent changes of the fluorescence images were found, for example precipitating hydrate phases, is the overall bad interpretability of the fluorescence microscopic data caused by the silica fume. Up to this point, it is not quite clear how far this issue may be overcome by improved preparation setups and microscopic methods in the future. Even if single silica particles could be resolved for example with confocal laser scanning microscopy (CLSM), the Brownian motion would lead to blurry images.

The experimental setups and conditions for the cement hydration of both used methods in this work are quite different. Thus, a quantitative comparison is not appropriate, but it could be shown that qualitative prediction via fluorescence microscopic analyses concerning the retarding effect of superplasticizers are plausible.

## 4. Conclusions

For the effective use of UHPC in various applications, well-adjusted systems are of major importance. Concerning special characteristics and requirements of UHPC mixing, it is essential to use optimized and balanced admixtures to face certain challenges. For example, silica fume is needed as pozzolanic and filling material but may cause strong incompatibilities with superplasticizers [35] and often requires high and thus expensive amounts of those admixtures. For less complex cementitious systems, a common and well investigated way to reduce the demand of superplasticizers is the delayed addition to the mixture [14], but for UHPC this method is not applicable due to the formation of indispersible agglomerates in the very early mixing period. As already mentioned, especially the complex system of UHPC is hard to simulate with theoretical approaches to predict the different effects of additives. As shown in this work, the effects caused by the used admixtures are strongly depending on their chemical structure and the composition of the cementitious system. The extreme retardation caused by APEG was quite surprising but coherent for both presented methods and the microscopic results support the idea of SP-covered and thus passivated reactive surfaces. The role of gypsum in the shown experiments is very interesting and arises some further questions. Different approaches may provide better understanding of the presented relations. From the microscopic point of view, the investigation of simplified systems is promising if pure clinker phases and defined mixtures with gypsum or hemihydrate are investigated. On the other hand, the setting of cement with different C_3_A content in combination with varying PCEs (structure and dosage) should be taken into account.

As a compromise between effort and research progress, the fluorescence microscopic method reveals information about the interactions of additives and mineral systems in real time to find the best adjustments for the versatile construction material.

## Figures and Tables

**Table 1:** Phases composition of used cement estimated by XRD and Rietveld refinement.

Phase	C_3_S	C_2_S	C_3_A	C_2_AF	Gypsum and Hemihydrate
Content [%]	61.3	15.9	3.9	13.9	3.3

**Table 2:** Oxide composition determined by XFA.

Oxide Content [wt%]	SiO_2_	Al_2_O_3_	Fe_2_O_3_	CaO	MgO	K_2_O	Na_2_O	P_2_O_5_	Others	LOI
Silica fume	95.6	0.1	0.1	0.5	0.3	0.7	0.2	0.1	1.0	1.4
OPC	21.5	3.7	4.3	64.3	0.9	0.4	0.3	0.2	2.7	1.7

**Table 3:** Composition of calorimetry samples.

SP	%bwob	SP Conc.	SP Total Mass [g]	Cement [g]	Silica Fume [g]	Water [g]	Total [g]
-	-	-	-	15	0	6	21
APEG	0.5	35 wt%	0.21	15	0	3.61	18.82
MPEG	0.5	40 wt%	0.19	15	0	3.63	18.82
APEG	1.2	35 wt%	0.51	15	0	3.42	18.93
MPEG	1.2	40 wt%	0.46	15	0	3.47	18.93
-	-	-	-	12.75	2.25	6	21
APEG	0.5	35 wt%	0.21	12.75	2.25	3.61	18.82
MPEG	0.5	40 wt%	0.19	12.75	2.25	3.63	18.82
APEG	1.2	35 wt%	0.51	12.75	2.25	3.42	18.93
MPEG	1.2	40 wt%	0.46	12.75	2.25	3.47	18.93
